# Effect of Scanning Speed on Properties of Laser Surface Remelted 304 Stainless Steel

**DOI:** 10.3390/mi13091426

**Published:** 2022-08-29

**Authors:** Yuanlong Chen, Xiang Li, Jinyang Liu, Yichi Zhang, Xuehui Chen

**Affiliations:** 1 School of Mechanical Engineering, Hefei University of Technology, No. 193, Tunxi Road, Hefei 230009, China; 2 School of Mechanical and Electrical Engineering, Anhui Jianzhu University, No. 292, Ziyun Road, Hefei 230601, China

**Keywords:** laser surface remelting, remelted layer, micromorphology, surface properties

## Abstract

In order to study the microstructure and properties of stainless steel after laser surface remelting, based on the theory of laser surface remelting, a simulation model of nanosecond-pulsed laser surface remelted stainless steel was established to study the evolution law of the Marangoni force of the molten pool during laser surface remelting. A single-lane laser remelting experiment was performed to study the variation of the scanning speed on the remelting width, roughness, and layer microtopography. The “S” scanning path was used to remelt the stainless steel surface to investigate the bonding force between the remelted layer and the substrate, the hardness, microscopic morphology, and corrosion resistance. The results show that the viscosity of the liquid metal in the molten pool increases with the increase of the scanning speed. Larger liquid viscosity and smaller surface tension temperature gradients promote a weaker flow of liquid metal, which reduces the velocity of the liquid metal flow in the molten pool. With the increase of scanning speed, the remelting width gradually decreases, but the roughness gradually increases. When the element content of Cr increases, the element content of Fe and O decreases. The surface is covered with an oxide film, the main components of which are oxides of Cr and Fe, the remelted layer is greater than that of the substrate, and the corrosion resistance is improved. Laser surface remelting technology can improve the structure and properties of 304 stainless steel.

## 1. Introduction

Stainless steel is widely used in the oil supply valve of the airport oil pipeline network due to its good mechanical properties and corrosion resistance, but it is vulnerable to fretting wear, corrosion, and other effects under service conditions, which affects its service life [1,2]. In order to improve the surface protection ability and prolong its service life under service conditions, surface strengthening methods are often used in engineering. At present, the techniques for changing the surface properties include chemical vapor deposition, physical vapor deposition, ion implantation, and laser surface modification. Therefore, a large number of scholars have carried out research on the surface modification of 304 stainless steel to improve the microstructure and properties.

Ghorbani et al. [3] used plasma-assisted chemical vapor deposition technology to prepare a Ta/TaN composite film on the surface of stainless steel to study its biocompatibility and corrosion resistance in the medical field. The corrosion rate in Ringer’s solution (compound NaCl injection) decreased by more than two orders of magnitude. Xiong et al. [4] deposited a Cr alloy layer on the surface of stainless steel using high-temperature chemical vapor deposition technology to explore the corrosion resistance of stainless steel protected by a Cr film layer in simulated seawater. The experimental results show that the deposition of Cr film on the surface of 316L stainless steel reduces pitting corrosion and crevice corrosion. In chemical vapor deposition, the deposition temperature is too high, which can easily cause changes in the substrate material and structure. The reaction source deposited during the reaction and the residual gas after the reaction have problems such as flammability, explosion, toxicity, and difficulty in local film deposition.

Kaliaraj et al. [5] prepared monoclinic and tetragonal ZrO_2_ ceramic coatings on the surface of 316L stainless steel by electron beam physical vapor deposition. The test results show that the coating has higher surface resistance and corrosion resistance than 316L stainless steel. Fu et al. [6] used pulsed bias arc ion-plating technology to deposit chromium nitride films with different composition ratios on stainless steel substrates, and studied their corrosion resistance in the fuel cell catholyte environment. The results show that the corrosion current density of the stainless steel after surface treatment in the electrolyte is reduced by an order of magnitude compared with that before the surface treatment, which significantly improves the service life of the stainless steel as a fuel cell bipolar plate. The physical vapor deposition method uses some special physical methods to excite molecules, atoms, or ions from the target substrate, and then go through a natural deposition or electric field deposition process. The disadvantage is that the bonding force between the coating film and the substrate is weak, and the coating material easily falls off the surface of the substrate, thereby increasing the corrosion rate of stainless steel.

Xu et al. [7] used the double glow cathode discharge technology to prepare a multi-component nano-alloy diffusion layer containing Al, Ta, Si, and other elements on the surface of stainless steel. In the mud corrosion and ultrasonic cavitation corrosion tests, the alloy diffusion layer showed more excellent resistance performance. Yetim et al. [8] implanted N element on the surface of stainless steel at different experimental temperatures and conducted electrochemical corrosion tests on the treated workpiece. The study found that with the gradual increase of the diffusion temperature, the diffusion layer rich in N element improved the corrosion resistance of the substrate. Ion implantation technology is generally used for the diffusion of elements with a smaller atomic radius, such as non-metallic elements such as C, B, and N, and can also be used for the diffusion of metal elements with a large atomic radius, such as Cr, Ni, and other elements. However, the relative atomic mass of these elements is relatively large and the acceleration ability in the electric field is limited. The movement resistance of metal ions after entering the substrate is greater and the diffusion distance is very short, which is difficult to meet the requirements of long-term service.

Pei et al. [9] comparatively studied the effect of laser remelting Q235 steel and laser thermal spraying Al85Ni8Y4Ce3 amorphous coating on corrosion performance in 0.5 mol/L NaCl solution. The test results show that a dense passivation film is formed during the laser remelting process, which inhibits pitting corrosion and improves corrosion resistance. Li Peng et al. [10] investigated the changes in the corrosion resistance of laser-remelted 304 stainless steel surfaces. The results show that the laser remelting treatment of 304 stainless steel surface can change its microstructure, refine grains, and improve its corrosion resistance. Khorram et al. [11] conducted laser cladding experiments on IN718 superalloy. The average hardness value of the cladding layer hardness is 1050 HV, which is about 2.5 times the hardness of the substrate. Shen et al. [12] prepared 431 stainless steel coatings with a cladding speed of 1.5–100 m/min, and studied the microstructure and corrosion resistance of the coatings. The results show that the higher the cladding speed, the smaller the dendrite size of the coating, the more uniform the composition, and the better the corrosion resistance.

Yuan et al. [13] prepared Ni45 coatings on 45 steel using conventional and ultra-high-speed laser cladding processes to study the microstructure and corrosion resistance of the coatings. The results show that the microstructures of the two coatings have the same growth law, but the microstructure of the high-speed laser cladding coating is smaller and denser. Taltavull et al. [14] performed the laser surface remelting of AZ91D magnesium alloy to explore the effect of laser power and scanning speed on the surface corrosion performance. The results show that with the increase of laser input energy, the corrosion resistance of the magnesium alloy surface is improved. Ha et al. [15] conducted laser remelting of magnesium alloy sheets and found that with the increase of scanning speed, the microhardness of the sheet increased and the elongation decreased. Laser surface remelting is one of the laser surface modification technologies. Without adding any metal elements, the surface is partially melted by a laser beam and then rapidly cooled to refine the surface of the metal material, eliminate pores and microcracks on the surface, and make the structure more dense, thereby improving the surface properties of the material.

The first three technical processes are more complicated, resulting in the higher cost of surface modification of 304 stainless steel. Laser surface remelting technology is a low-cost surface modification technology. Therefore, in order to improve the surface structure and performance of 304 stainless steel, based on the principle of laser remelting, a nanosecond pulsed laser is used to remelt 304 stainless steel.

Moreover, it can be seen from the above review that although scholars have carried out a lot of research on surface modification technology, most scholars only establish the temperature field model of laser remelting, but have not established the physical field model of the Marangoni force effect when laser remelting stainless steel. The bonding strength between the remelted layer and the substrate after laser remelting stainless steel was not studied and the surface roughness of laser remelted stainless steel was not studied. Therefore, in this paper, the Marangoni force effect model was first established to analyze the influence of the Marangoni effect on the remelted layer during the laser surface remelting of stainless steel, and then the microstructure, roughness, hardness, bonding force, and corrosion resistance of the remelted layer were studied.

## 2. Laser Remelting Principle and Model Establishment

### 2.1. Principle of Laser Remelting

**Figure 1:**
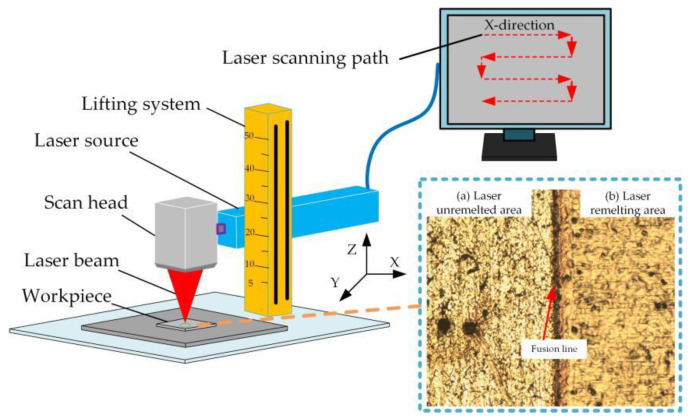
Principle of laser surface remelting.

Laser surface remelting uses a high-energy laser beam to continuously scan the surface of the material to form a thinner melted layer. The metal liquid in the molten pool is rapidly cooled and solidified by the heat conduction of the substrate, thereby refining the alloy structure and reducing the segregation, which improves the comprehensive performance of the workpiece surface [16,17]. Laser surface remelting can obtain different microscopic features on the surface of the material, changing the surface morphology, composition, hardness, and corrosion resistance of the substrate. The principle of laser surface remelting is shown in Figure 1. Applying this technology to the surface modification of 304 stainless steel can greatly improve the comprehensive performance of the 304 stainless steel surface.

**Figure 2:**
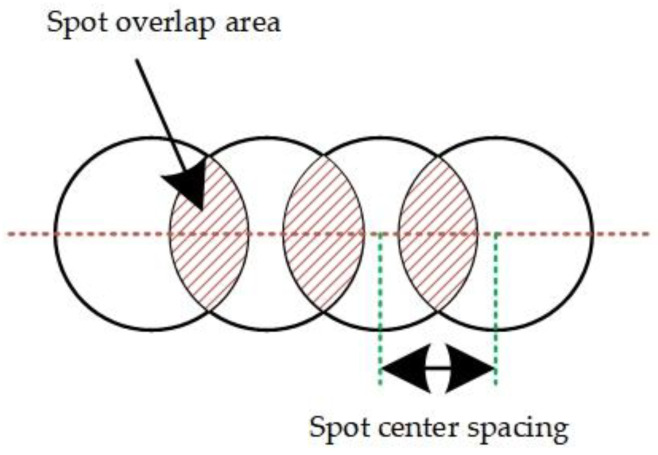
Spot overlap diagram.

In laser surface remelting, the remelted layer morphology is related to the laser spot overlap rate and the number of pulses deposited on the material surface. One laser pulse can form a light source on the surface of the sample and the two light spots will overlap during the scanning process, as shown in Figure 2. The overlap ratio of the spots and the number of pulses per unit area can be changed by the scan speed.

Because the spot is extremely small (50 μm) and the pulse duration is nanosecond level (10^−9^ s), it is much smaller than the adjacent pulse interval (10^−5^ s). Therefore, in order to facilitate the calculation, the light spot is approximated as a square, the reciprocal of the repetition frequency is approximated as the time interval of two adjacent pulses, and the calculation formula of the light spot overlap ratio is obtained [18,19].

(1) $$\varphi =\frac{d-v/f}{d}=1-\frac{v}{d\cdot f}$$

Here, *φ* is the spot repetition rate; d is the spot diameter; *v* is the scanning speed; *f* is the laser repetition frequency; and *v*/*f* is the spot center spacing.

During the interaction between the laser and the stainless steel, the laser spot overlap ratio determines the amount of laser energy deposited on the stainless steel surface along the laser scanning direction. When the laser repetition rate and the spot size are constant, the spot overlap rate is only related to the scanning speed. It can be seen from Equation (1) that with the increase of the scanning speed, the distance between the centers of adjacent spots increases, the overlap of the spots decreases, the number of pulses per unit area decreases, and the laser energy deposited on the stainless steel surface decreases. Depending on the cumulative effect of the multi-pulse ablation, the scanning speed induces the formation of different microstructures and properties on the surface of the material.

### 2.2. Model Establishment

The surface heat source model with Gaussian distribution can better reflect the laser surface remelting state, and the laser energy density equation is [20,21]

(2) $$q(x,y,t)=\frac{2\eta P}{f\tau \pi r^{2}}\exp\left(-2\frac{\left(x^{2}-x_{0}^{2}\right)+\left(y^{2}-y_{0}^{2}\right)}{r^{2}}\right)\cdot g\left(t\right)$$

(3) $$g(t)=\left\{ \begin{array}{l} \exp\left[-4\ln2{\left(\frac{t-2\tau }{\tau }\right)}^{2}\right],jT_{c}\leq t<4\tau +jT_{c}\\ 0,\tau +jT_{c}\leq t<\tau +\left(j+1\right)T_{c} \end{array}\right.j\subset N$$

where *q* (*x*, *y*, *t*) is the laser energy density function; *P* is the laser power; *η* is the laser absorption rate of the material; *r* is the laser spot radius; *f* is the laser repetition frequency; *τ* is the laser pulse width; *j* is a natural number; *t* is the heat source loading time; the function *g*(*t*) is a periodic pulse function; *x* and y represent the two directions of the Cartesian coordinate system, respectively; and *n* = 1, 2, 3, ...; *T_c_* is the pulse period.

**Figure 3:**
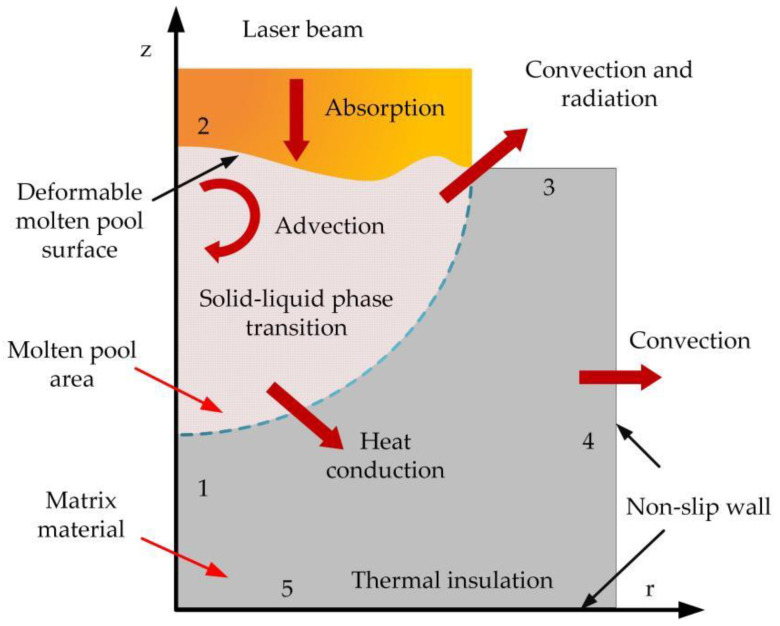
Laser remelting physical mold.

The physical model of laser surface remelted 304 stainless steel is established in the COMSOL Multiphysics, as shown in Figure 3, in which the physical properties and boundary conditions of the stainless steel are set in Table 1 and Table 2, respectively.

## 3. Experimental Equipment and Methods

The research object selected was 304 stainless steel, and it was processed by cutting, grinding and polishing, ultrasonic cleaning, drying, and sealing to obtain a 10 mm × 10 mm × 3 mm surface smooth and flat specimen to be processed. The average power of the laser in this paper is 20 W, the fixed pulse width is 100 ns, the spot diameter is 50 μm, the repetition frequency is 70 kHz, and the scanning interval is selected to be 0.01 mm. After several test experiments, the scanning speed of the laser was set to 10 mm/s, 20 mm/s, 30 mm/s, and 40 mm/s, respectively.

**Figure 4:**
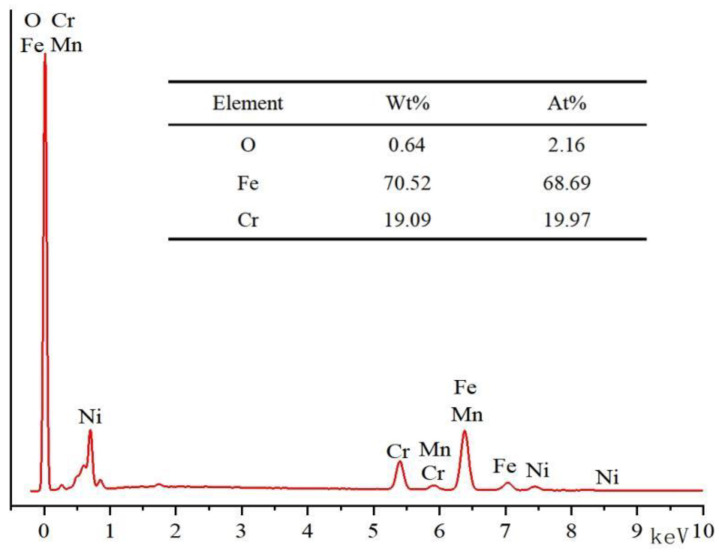
Main elements of the substrate.

The main elements and EDS of the substrate before laser surface remelting are shown in Figure 4. It can be seen from Figure 4 that there are few oxides on the initial surface of the stainless steel, which is conducive to analyzing the change of element content and the amount of oxide formation after laser surface remelting.

The surface morphology and microstructure of the specimens after laser surface remelting were observed by scanning electron microscope, and the types and contents of the chemical elements on the surface were analyzed by EDS. The roughness of the remelted layer was observed by a 3D confocal microscope. A fixed-target X-ray diffractometer was used to measure the XRD of the stainless steel before and after laser surface remelting. The hardness of the remelted layer was tested by an HVS-50ZCTC microhardness tester; the test load was 50 kgf, the load time was 10 s, and the distance between each measurement point was 0.8 mm. The polarization curve of the remelted layer was measured by a CHI600E series electrochemical analyzer. The reference electrode was a saturated calomel electrode, the auxiliary electrode was a platinum electrode, and the voltage scanning range was −1 V to 0.5 V. The scanning rate was 5 mV/s.

## 4. Analysis of Simulation Results

**Figure 5:**
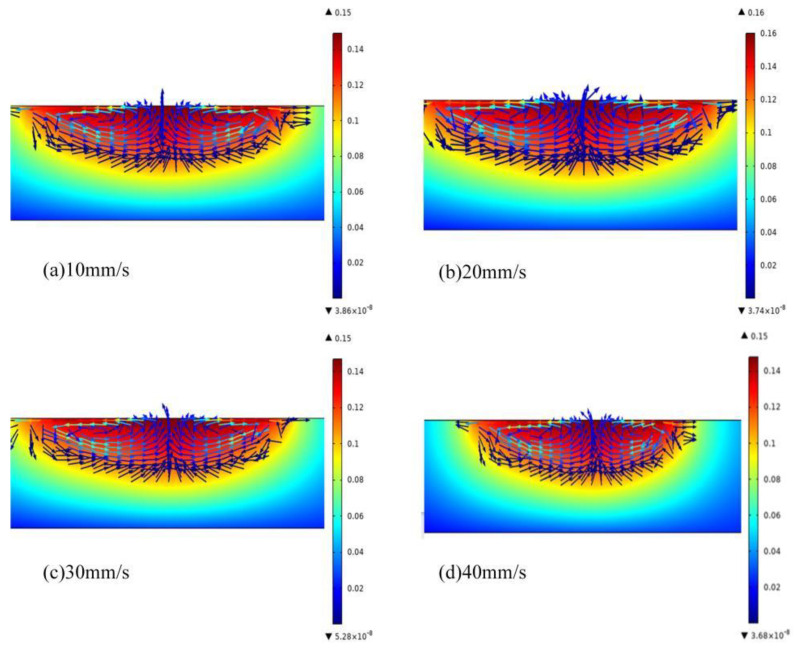
Distribution of molten pool and Marangoni force at different scanning speeds, (**a**) 10 mm/s; (**b**) 20 mm/s; (**c**) 30 mm/s; (**d**) 40 mm/s.

The other conditions of the laser remained unchanged, and the scanning speed was set to 10 mm/s, 20 mm/s, 30 mm/s, and 40 mm/s to simulate the molten pool and Marangoni effect. The simulation results are shown in Figure 5.

It can be seen from Figure 5 that with the increase of the laser scanning speed, the maximum flow rate of the liquid metal in the molten pool first gradually increases, and then decreases. It is analyzed that when the laser scanning speed is 10 mm/s, the stainless steel surface absorbs more energy. Although the Marangoni effect [24] is strong, the amount of melting on the surface of the substrate is large at this time, so that more molten material on the surface of the stainless steel accumulates on the machined surface, hindering the flow of the internal molten pool. As the laser-scanning speed increases, the interaction time between the surface of the metal material and the laser heat source increases, resulting in a cumulative decrease in the heat and a continuous drop in the temperature of the molten pool, which will reduce the surface temperature gradient of the molten pool. The smaller the temperature of the molten pool, the greater the viscosity of the liquid metal in the molten pool. The larger liquid viscosity and smaller surface tension temperature gradient promote the weak flow of liquid metal, which will gradually reduce the speed of the liquid metal flow in the molten pool. When the laser-scanning speed continues to increase to 40 mm/s, the laser energy acting on the surface of the material is less, the modification effect on the surface of the material is weak, the melting amount of stainless steel is less, and the flow inside the molten pool slows down.

## 5. Analysis of Experimental Results

In the laser surface remelting of stainless steel experiment, the experimental environment, experimental materials, processing parameters of the nanosecond laser, and the state of the material will affect the results of the experiment. In this paper, the change law of the surface microstructure and properties is studied by changing the scanning speed of the laser. According to the experimental principle in Section 2, it can be seen that the scanning speed will affect the spot overlap rate and then change the amount of laser energy deposited on the surface of the material.

### 5.1. Micro-Dimensional Changes of Stainless Steel Samples at Different Scanning Speeds

**Figure 6:**
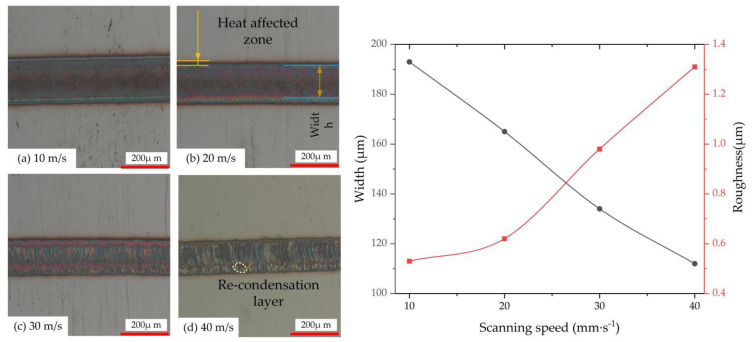
Micro-size changes of stainless steel samples at different scanning speeds, (**a**) 10 m/s; (**b**) 20 m/s; (**c**) 30 m/s; (**d**) 40 m/s.

The scanning speed affects the spot overlap ratio, which, in turn, affects the laser energy. Figure 6 shows the single-channel remelting width and roughness observed by the metallographic microscope and 3D laser confocal microscope.

It can be seen from Figure 6 that with the increase of scanning speed, the width of remelting gradually decreases, but the roughness gradually increases.

When the laser-scanning speed is small, as shown in Figure 6a, the distance between the centers of adjacent spots is small, the overlap of the spots is high, and the laser energy deposited on the stainless steel surface is high, which makes the surface of the metal material melt more. Under the action of the Marangoni force effect [25,26], the molten material inside the molten pool is driven to flow, so that the machined surface is relatively flat and has low roughness. However, the effect of the laser energy is greater than that of the Marangoni force, which makes the surface of the stainless steel melt more and results in a larger width.

The large energy density leads to a large temperature gradient of surface tension, which makes the solid–liquid level of the molten pool fluctuate. In addition, the laser-scanning speed is small, and the liquid phase metal exists for a very short time, which causes the viscosity of the molten pool to rise sharply and reduces the wettability of the liquid-phase metal. At this moment, the liquid metal in the molten pool flows from the high-temperature area to the low-temperature area. The temperature of the molten pool and the surface tension are negatively correlated, so the surface tension of the molten pool is larger at this time, and the driving force of the molten pool is mainly dominated by the surface tension.

Figure 6b shows that when the laser-scanning speed increases to 20 mm/s, although the energy density decreases, the maximum flow velocity in the molten pool increases to 0.16 m/s (Figure 5b). The larger temperature gradient in the molten pool forms a larger Marangoni convection phenomenon, which is beneficial to the heat and mass transfer of the materials in the molten pool, resulting in a wider molten pool morphology. As the scanning speed increases to 40 mm/s, the distance between the centers of adjacent spots increases, which reduces the number of pulses per unit area and the laser energy deposited on the stainless steel surface, and the remelting width decreases. Due to the reduced overlap of the light spots, the latter light spot can melt the area that was not melted by the previous light spot in time, and the surface roughness is larger.

### 5.2. Micromorphology of Stainless Steel at Different Scanning Speeds

**Figure 7:**
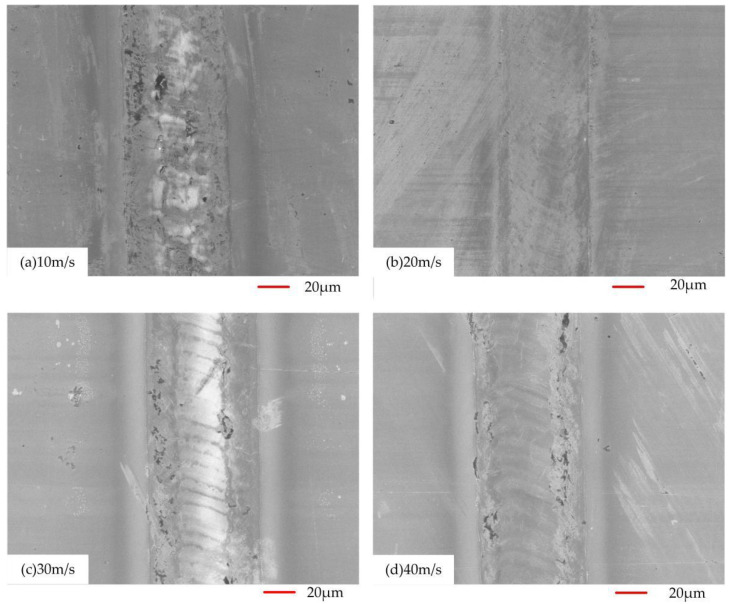
Micromorphology of the remelted layer at different scanning speeds, (**a**) 10 m/s; (**b**) 20 m/s; (**c**) 30 m/s; (**d**) 40 m/s.

In order to further observe the microscopic morphology after remelting, the remelted layer was observed by scanning electron microscope. Figure 7 shows the scanning electron microscope images of the specimens at different scanning speeds.

It can be seen from Figure 7 that after laser surface remelting, concave and convex patterns with different distributions are formed on the surface of the stainless steel, and the depth of the concave patterns and the size and morphology of the convex patterns are different at different scanning speeds. The surface roughness shows a gradually increasing trend during the change of the laser scanning speed within 10–40 mm/s. When the scanning speed reaches 20 mm/s, the surface is relatively flat without cracks and exhibits “fish scale”-like protrusions.

When the scanning speed is 5 mm/s, the bulge distribution is sparse and irregular and the shape is irregular. A clear demarcation between the protrusions and depressions cannot be seen from Figure 7a. Due to the serious local energy accumulation on the surface of the material, the material that melted and solidified before and after the laser action process cannot form a convex structure with a certain shape, and the flow in the field is chaotic, resulting in a small surface roughness and more obvious cracks. It is analyzed that when the scanning speed is small, the spot overlap rate is higher and the material absorbs more laser energy, which produces a stronger ablation effect. The surface of the substrate is seriously ablated and material debris appears in the remelting layer, which causes the material to vaporize at an excessively high temperature, destroying the formed remelted layer. Moreover, due to the large temperature gradient in the cooling process of the laser remelted layer, the internal thermal stress of the material is affected by the imbalance, which makes microcracks appear in the remelted layer.

Until the scanning speed reaches 30 mm/s, the obvious demarcation between the convex and concave surface of the specimen can be observed in Figure 7c. The protrusions on the surface of the material begin to form according to the morphology of “fish scales”, and no obvious cracks are observed on the surface. At this time, due to the low overlap rate of the light spot per unit time, the local energy accumulation is less on the surface of the specimen. The latter spot fails to melt the area that the previous spot did not melt, thus forming a convex structure with a certain shape and a large surface roughness.

**Figure 8:**
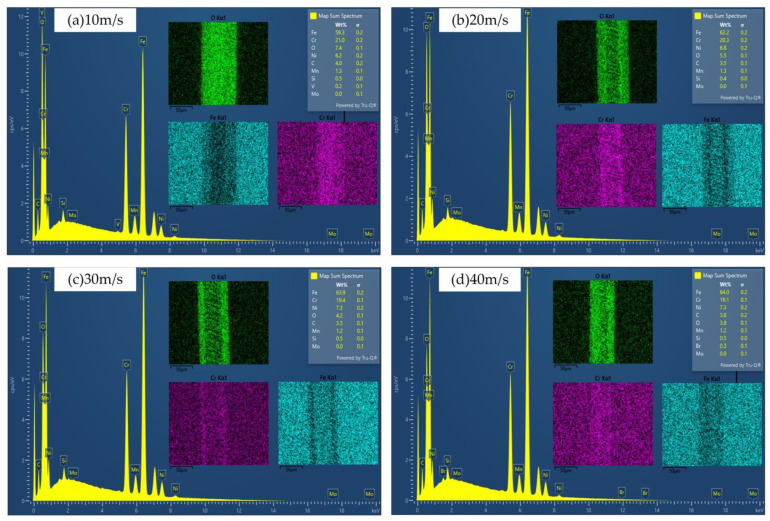
EDS test of remelted layer composition, (**a**) 10 m/s; (**b**) 20 m/s; (**c**) 30 m/s; (**d**) 40 m/s.

Figure 8 shows the EDS results of the chemical composition of the material surface after laser surface remelting. With the increase in the laser-scanning speed, the Cr content decreased from 21% to 19.1%, the O content decreased from 7.4% to 3.8%, and the Fe content decreased from 59.3% to 64.0%. It can be inferred from this that oxidation reaction occurred on the stainless steel surface after laser scanning. The increase in the laser-scanning speed leads to the decrease in the laser energy and the weakening of the oxidation reaction. Although Cr oxides are reduced, the main component of the substrate is Fe element, which still undergoes oxidation reaction, and the content of Fe oxides increases. The severity of the oxidation reaction has a great relationship with the scanning speed, or the ablation of the surface. When the scanning speed is small, the spot overlap rate and the energy deposited on the stainless steel surface are large, and the degree of oxidation reaction on the surface of the material in the air environment is more severe.

Compared with the experiment and simulation, it can be seen that when the scanning speed is 20 mm/s, the inside of the molten pool mainly flows to both sides, which is beneficial to the mass transfer and heat transfer inside the molten pool and the formation of a dense modified layer.

### 5.3. Laser Surface Remelting with “S” Scanning Path

**Figure 9:**
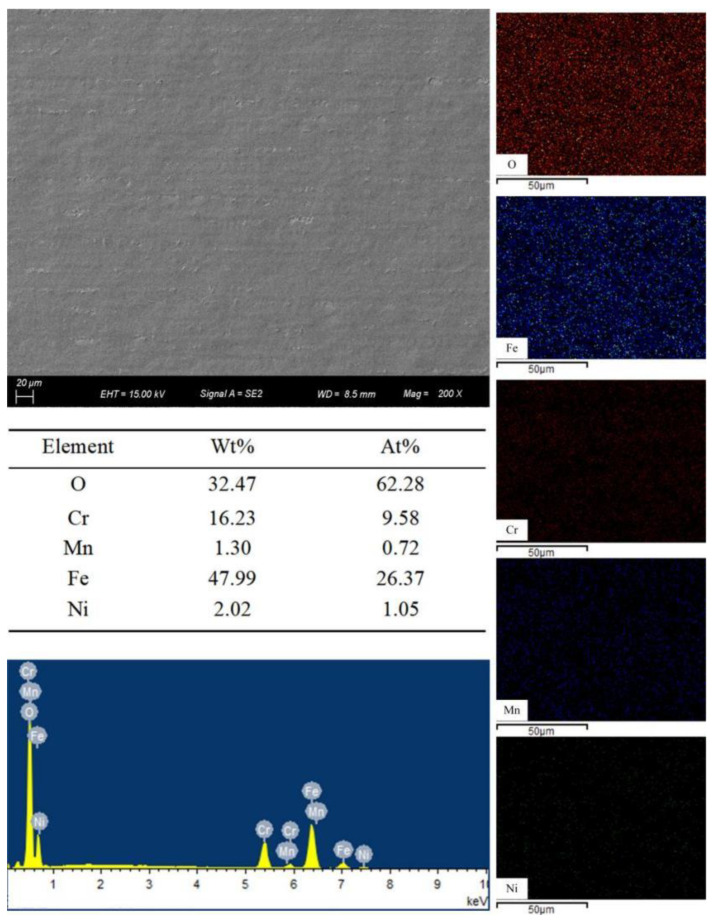
Micromorphology and EDS analysis after laser surface remelting with “S” scanning path.

The laser surface remelting parameters were reasonably chosen to remelt the stainless steel according to the laser scanning path shown in Figure 1. A scanning electron microscope was used to observe the remelted layer. Figure 9 shows the surface microstructure and element distribution of the remelted layer.

It can be seen from Figure 9 that the surface of the remelted layer presents a relatively obvious microscopic remelted morphology, and the surface modification effect of the stainless steel is better. The degree of vaporization of the material is relatively weak, the material on the surface of the substrate is melted, and the surface of the material is covered by a thin layer of molten oxide. The distribution of each element on the surface of the remelted layer indicates that the molten oxide layer mainly consists of oxides of Cr and Fe. Due to less material melting and less material residue, the surface of the remelted layer is smooth, and the surface quality of the remelted layer is good and free of microcracks. It is analyzed that when the laser reaches the stainless steel surface, the laser energy is basically absorbed and scattered, and the material in the treated area is melted and vaporized. Combined with Figure 5, it can be seen that the Marangoni effect drives the flow and redistribution of materials inside the molten pool. During the cooling process, the microscopic region on the surface of the material exhibits relatively regular topographical features.

**Figure 10:**
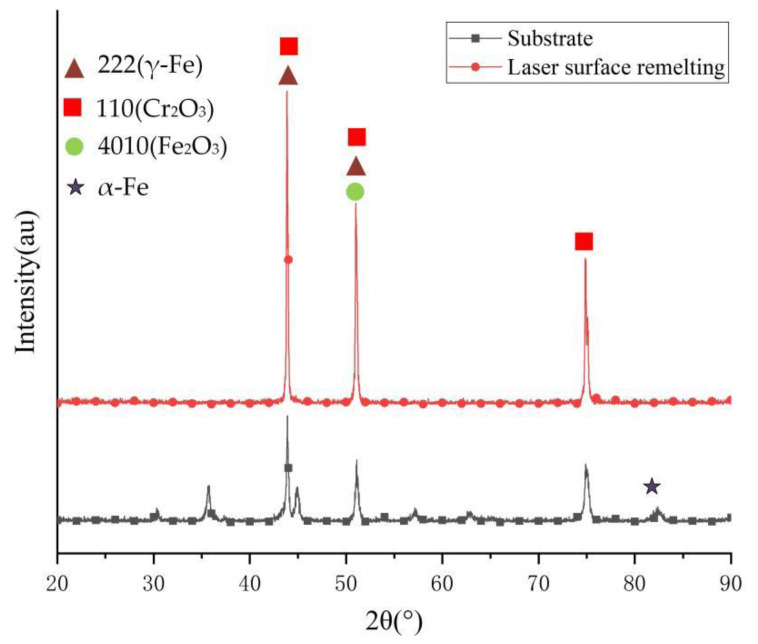
XRD of 304 stainless steel before and after laser surface remelting.

An X-ray diffractometer was used to measure the elemental composition of the stainless steel remelted layer, as shown in Figure 10. It can be seen from Figure 9 that a layer of oxides covering the metal surface is formed on the stainless steel after laser surface remelting, mainly chromium and iron oxides—the transformation from α-Fe to γ-Fe phase.

**Figure 11:**
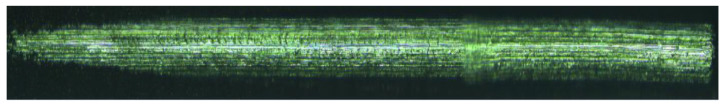
Surface topography after scratch testing.

Adhesion is the mutual bonding performance between the remelted layer and the substrate and is an important index to measure the performance of the remelted layer. A scratch tester was used to test the adhesion, which is 22.3 N, and the surface morphology after the scratch test is shown in Figure 11.

**Figure 12:**
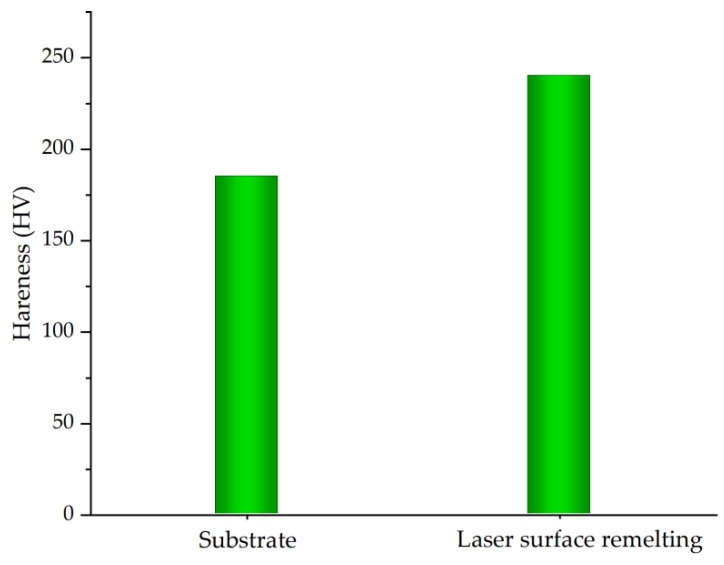
Surface hardness of stainless steel before and after laser remelting.

An HVS-50ZCTC microhardness tester was used to test the hardness of the remelted layer. In order to ensure the accuracy of the test data, three tests were carried out on different positions of the sample surface, and the average value was taken to represent the microhardness of the remelted layer. In this paper, Vickers hardness is mainly used to evaluate the hardness of the remelted layer. The microhardness test results are shown in Figure 12. The hardness of the substrate is 185.2 HV, and the hardness after laser surface remelting is 240.3 HV. It can be seen that the laser surface remelting technology can improve the surface hardness of stainless steel.

**Figure 13:**
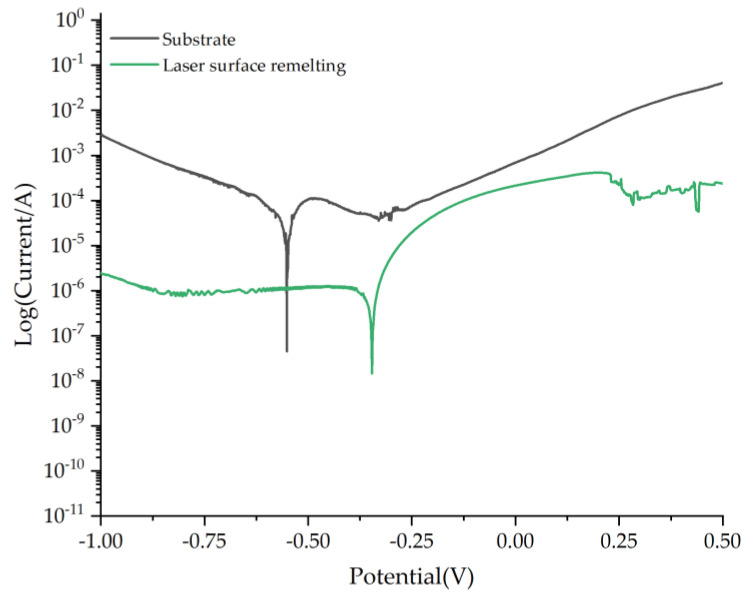
Potentiodynamic polarization curves of stainless steel remelted layer.

Generally, the self-corrosion potential is used to characterize the corrosion resistance of materials. The higher the self-corrosion potential of the remelted layer, the slower the internal penetration and diffusion, the slower the corrosion tendency and corrosion reaction, and the better the corrosion resistance. The electrolyte used in the electrochemical test was NaCl solution. The polarization curves of the substrate and the remelted layer were tested using an electrochemical three-electrode system. The voltage sweep range is −1 V–0.5 V and the sweep rate is 5 mV/s. Figure 13 is the measured polarization curve.

It can be seen from Figure 12 that the self-corrosion potentials of the stainless steel substrate and after laser remelting are −0.551 V and −0.346 V, respectively, and the corrosion resistance is improved. As the applied voltage increases, the remelted layer on the surface of the material is gradually dissolved and broken, the stainless steel substrate elements undergo electrochemical reactions, and the corrosion current density increases sharply.

Combined with Figure 8, it can be seen that during the laser surface remelting process of stainless steel, a dense remelted layer is formed on the surface, which is not easily eroded by anions in the NaCl solution and hinders the electrochemical reaction of the material.

## 6. Conclusions

In this paper, a nanosecond-pulsed laser is used to remelt 304 stainless steel to study the effect of laser scanning speed on the microstructure, mechanical properties, and corrosion resistance of the remelted layer on the surface of 304 stainless steel. The main conclusions are as follows:

(1) In the laser single-pass remelting, the surface roughness gradually increases with the laser scanning speed. When the scanning speed is 10 mm/s, the overlap rate of the light spot per unit time is high, and the latter light spot can melt the area that the previous light spot did not melt in time. The fluid flow inside the molten pool is driven by the Marangoni effect, and the surface of the remelted layer is relatively smooth. When the scanning speed is 20 mm/s, the surface of the remelted layer is relatively flat, without microcracks and “fish scale”-like protrusions. At this time, the spot overlap rate, the laser energy, and the material-melting rate reach a dynamic balance, which can not only melt the unmelted area of the previous spot, but also prevent microcracks and serious ablation, which makes the remelted surface smoother and flat. When the scanning speed is increased to 30 mm/s, the surface of the remelted layer begins to exhibit “fish scale”-like protrusions. When the scanning speed continues to increase to 40 mm/s, the “fish scale”-like protrusions are more serious and the roughness is larger.

(2) When laser surface remelting with an “S” scan path, the oxygen content in the remelted surface increases significantly. A layer of oxides is formed, covering the metal surface, mainly consisting of chromium and iron oxides. There is basically no slag accumulation or microcracks in the remelted layer, and the bonding strength with the substrate is good. The adhesion between the remelted layer and the substrate is 22.3 N.

(3) Laser surface remelting hardens the surface of the substrate and improves the corrosion resistance of the substrate. The substrate hardness is 185 HV. After laser surface remelting, the remelted layer hardness is greater than that of the substrate and the maximum value is 248.9 HV, which is 55.1 HV higher than that of the substrate. The self-corrosion potential of the remelted layer is −0.346 V, which is a positive shift of 0.205 V compared with the self-corrosion potential of the substrate, and the corrosion resistance is improved.

Laser surface remelting technology can not only improve the surface structure of stainless steel, but also improve the surface hardness and corrosion resistance.

## Figures and Tables

**Table 1:** Physical properties of 304 stainless steel [22,23].

Nomenclature	Value
Specific heat of liquid phase, *c* (J·kg^−1^·K^−1^)	780
Latent heat of fusion, *H* (J·kg^−1^)	2.47 × 10^5^
Latent heat of vaporization, *H_v_* (J·kg^−1^)	6.34 × 10^6^
Thermal conductivity of liquid phase, *k_l_* (W·m^−1^·K^−1^)	22
Density of liquid phase, *ρ_l_* (kg·m^−3^)	6900
Dynamic viscosity, *μ* (kg·m^−1^·s^−1^)	0.006
Solidus temperature, *T_s_* (K)	1670
Liquidus temperature, *T_m_* (K)	1727
Boiling temperature, *T_v_* (K)	3200
Ambient temperature, *T_∞_* (K)	300
Thermal expansivity, *β* (K^−1^)	4.95 × 10^−5^
Surface tension at *T_m_*, *σ_m_* (N·m^−1^)	1.2
Surface tension gradient, *A_σ_* (N·m^−1^·K^−1^)	−0.43 × 10^−3^

**Table 2:** Boundary condition settings.

Physical Field	Physical Meaning	Border Number	Boundary Conditions
Heat transfer field	Laser irradiation	2	Heat flux
	Natural convection	2,3,4	Convection
	Surface-ambient radiation	2,3,4	Diffusing surface
	Adiabatic	5	Thermal insulation
Fluid field	Surface Tension	2	Weak contribution
	Marangoni effect	2	Marangoni effect
	Wall	3,4,5	Non-slip wall
Move the grid	Fixed boundaries	1,3,4,5	Grid displacement
	Free deformation	2	Mesh normal velocity

## Data Availability

Not applicable.
